# Putting down the revolt: Enactivism as a philosophy of nature

**DOI:** 10.3389/fpsyg.2022.948733

**Published:** 2022-10-21

**Authors:** Russell Meyer, Nick Brancazio

**Affiliations:** School of Humanities and Social Inquiry, University of Wollongong, Wollongong, NSW, Australia

**Keywords:** enactivism, explanation, paradigm, philosophy of nature, embodied cognition, social cognition

## Abstract

Enactivists frequently argue their account heralds a revolution in cognitive science: enactivism will unseat cognitivism as the dominant paradigm. We examine the lines of reasoning enactivists employ in stirring revolt, but show that none of these prove compelling reasons for cognitivism to be replaced by enactivism. First, we examine the *hard sell* of enactivism: enactivism reveals a critical explanatory gap at the heart of cognitivism. We show that enactivism does not meet the requirements to incite a paradigm shift in the Kuhnian sense—there is no internal crisis in cognitivism. Nor does it provide inherently better explanations of cognition as some have claimed. Second, we consider the *soft sell* of enactivism: enactivism provides a more attractive, parsimonious, or clear-eyed lens on cognition. This move proves to boil down to a misunderstanding of how theories are selected in science. Instead we lend support to a broader and more desirable way to conceive of enactivism, the recent proposal that enactivism is a *philosophy of nature*. We explain how a philosophy of nature does more than support a single research paradigm by integrating scientific questions into a cohesive picture.

*“The big problem I have about enactivism is figuring out what it is.”*—Ned Block

## Introduction

Contemporary cognitive science seeks to find and explain the internal mechanisms that, in a similar fashion to human-designed computers, represent and process our sensory inputs, thought processes, and produce our motor outputs. The theory of enactivism, developed from the work of Maturana and Varela (1980), eschews this computational analogy of the mind and instead looks to build a theory of cognition tied to fundamental organizational biological processes. Now a fairly cohesive research programme after further development by Varela et al. (1991), enactivism posits a fundamental linkage between biological organization and cognitive processes. The research program of enactivism, drawing also from the embodied, action-oriented phenomenology of Merleau-Ponty (2012), takes cognitive processes to be necessarily world-involving, as the organism’s morphology, history, and the biological conditions of its persistence establish what the organism is able and aiming to do. In proposing these sweeping changes to how we understand the connection between living and cognition, the enactivist literature is often suggestive of a worldview, of overarching commitments and principles that differ greatly from those held by classical cognitivist science.

Contemporary enactivism “emphasize[s] the role of the dynamical coupling of brain–body–environment” (Di Paolo, 2018, p. 3). Enactivists “view the organism as a self-organizing, autonomous, autopoietic system” (Chemero, 2009, p. 152) and they “insist that biological aspects of bodily life, including organismic and emotion regulation of the entire body, have a permeating effect on cognition, as do processes of sensorimotor coupling between organism and environment” (Gallagher, 2017, p. 7). So described, enactivists make a statement of intent for a new approach to studying cognition. Statements of intent, though, are potentially vague about the specifics of what enactivism means beyond a certain angle on cognition. This picture is many things to many people: a heterodox alternative to traditional cognitive science, a remonstration of analytic philosophy of mind with the phenomenological tradition, an overarching story that explains the continuity of life and mind. What might jump out from this characterization is both its breadth, and its indeterminacy. Is enactivism the synthesis of divergent perspectives on the mind, an empirical program, the natural successor to cognitivism, all of the above, or something else?

The received view amongst enactivists seems to be that enactivism is primarily an alternate research paradigm for cognitive science. The stronger iteration of this view is that enactivism is not merely an explanatory alternative to cognitivism, but provides superior explanations when compared to its rival and thereby supersedes it (Hutto and Myin, 2013, 2017; Di Paolo et al. 2018). A weaker iteration is that enactivism is, rather than superior on some strict criteria of explanatory power, simply a more attractive option when compared to cognitivism, which has undesirable qualities (Di Paolo et al. 2017). We will argue that neither of these approaches heralds a coming enactive revolution, and that the critiques present in these approaches do little harm to cognitivism. However, we are not arguing, as many already have, that enactivism therefore ought to integrate itself with its rivals, give up its commitments (or smooth them over as mere expository or semantic disagreements) and come in from the cold back to mainstream cognitive science (Miłkowski et al., 2018).

Rather than backing the weaker reading of enactivism as an alternative research paradigm, we offer support for a more visionary alternative. Drawing on discussions by Godfrey-Smith (2001, 2014), and Gallagher (2017), we propose that a better way to think of enactivism is as a *philosophy of nature*. As a philosophy of nature, the breadth of concerns taken up in the enactivist literature address more than a single research paradigm. The proposals of enactivism aim not at replacing cognitivism as a research paradigm, but its underpinning (and more broadly pervasive) commitments.

In the following section, we will examine claims that enactivism is a research paradigm that will displace cognitivism through superior explanatory power, a move we call the *hard sell* of enactivism. Following Steiner (2019), we will argue that there is no such impending paradigm shift. We evaluate competing views on social cognition as an example of the explanatory adequacy of both cognitivism and enactivism, finding that the literature at this point provides no reason to favor one explanation over the other except for one’s own pre-existing commitment to either camp. In section 3 we evaluate the *soft sell* of enactivism, which pitches enactivism as a more intuitive or parsimonious way to understand cognition compared to cognitivism. We similarly find this approach to be unsuccessful, since its success hinges on one already holding enactive commitments. In section 4 we turn to discuss an alternative aim for enactivism: to serve as a philosophy of nature rather than a proposal for a scientific paradigm, an undertaking which we see as more achievable, more genuinely revolutionary and more aligned with the existing enactivist project.

## Is the sky falling? The hard sell

Talk of representation wars and cognitive revolutions is ubiquitous in the philosophy of cognitive science literature. The claim that contemporary enactivism views itself as a rival paradigm to cognitivism is fairly trivial, as it is mentioned pervasively in the literature: “There is a small but growing community of researchers spanning a spectrum of academic disciplines which are united in rejecting the still dominant computationalist framework in favor of the late biologist’s Francisco Varela’s *paradigm* of enaction” (Froese and Di Paolo, 2011, p. 1, emphasis added). Contemporary enactivists have warned us that “[r]evolution is, yet again, in the air” (Hutto and Myin, 2013, p. 1), that we are “in the midst of a major scientific revolution, properly described as a paradigm shift” (Gibbs, 2011, p. 82), and that “[t]he revolutionary narrative that has always accompanied enactivism is now warming up” (Barandiaran, 2017, p. 409). The “revolutionary ambitions” (Steiner, 2019, p. 4) of enactivism seem to rest on the idea that cognitive science has an impending paradigm shift on the horizon, one that will find enactivism replacing cognitivism as the dominant paradigm. This kind of talk has been buoyed by those criticizing enactivism or its more radical tenets. For example, Michael Wheeler, in detailing what would be needed for 4E to have a “truly paradigm-shifting result” (Wheeler, 2017, p. 2), has gone so far as to draw a comparison between the theses proposed by enactivists and the ‘April Theses’ delivered by Lenin at meetings of the *All-Russia Conference of Soviets of Workers’ and Soldiers’ Deputies* in 1917.

What is it about the enactive framework, though, that makes this talk of revolutions and paradigm-shifts appropriate? Should we understand enactivism as a rival *paradigm* to cognitivism? On Kuhn’s well-known account (Kuhn, 1962/2012) a paradigm refers to the shared commitments that enable the practice of a particular research program, exemplified by “universally recognized scientific achievements that for a time provide model problems and solutions to a community of practitioners” (Kuhn, 1962/2012, p. iii). A paradigm defines a field of research where it holds sway: it “is what the members of a scientific community, and they alone, share” (Kuhn, 1974, p. 294). Kuhn deems the general consensus about these shared commitments amongst scientists in a particular field *normal science*. This is when things are moving along more or less unproblematically, and where the practice of science under this paradigm is able to forge ahead (generate research) without running into any major holes or explanatory issues. Computational cognitive science is one such research paradigm: it has been the dominant explanatory framework for decades, it overthrew behaviorism as its paradigmatic predecessor, and it has been profoundly useful for conducting empirical research and designing testing procedures.

The appropriateness of the term “paradigm shift” to describe the endgame of enactivism has been challenged recently by Steiner (2019). Steiner points out that a paradigm shift results from the persistent failures of the existing paradigm to produce solutions to problems generated from within the paradigm. This leads to a crisis, followed by a scientific revolution when a succeeding paradigm is adopted: “A scientific revolution, for Kuhn, is radical: it does not consist in a change of observations, results and theories; it is a conceptual change in virtue of which the meaning of terms is drastically changed, but also a foundational change. New principles, laws and definitions are introduced” (Steiner, 2019, p. 7). Enactivists are certainly aware of the drastic and concrete changes needed to set up such an alternative: enactivism “would be of limited interest if it were only a collection of speculative arguments” (Di Paolo et al., 2017, p. 21). Much recent work has been aimed to formulate conceptual posits “in terms that allow for them to be tested, improved, and, if necessary, rejected by scientific standards” (Di Paolo et al., 2017, p. 21). Having set themselves up as a non-representational^1^ or anti-cognitivist framework, a counterpoint to the mainstream, enactivists have set their sights on producing a working empirical research program (i.e., Buhrmann et al., 2013; Buhrmann and Di Paolo, 2014; Rucinska and Reijmers, 2014; De Jaegher et al., 2017; Valenzuela-Moguillansky et al., 2021).

Assembling a paradigm is one thing, and bringing about a paradigm shift is another. In order to judge that a paradigm shift is nigh, computationalism must be on the verge of abandonment due to its inability to solve problems regarded as acute *from within the paradigm itself* (Kuhn, 1962/2012; Steiner, 2019). However, contemporary cognitive science suffers no inability to continue articulating new problems and investigating their solutions. The classical cognitivist paradigm is not debilitated by its reliance on computation and representations in a way that would generate wide-spread insecurity (Steiner, 2019, p. 8). While enactivists often point to differences between definitions of representation as indicating a lack of consensus amongst computationalists, a flexible definition is not in itself the kind of paradigm-weakening problem that enactivists seem to think it is; its numerous definitions can equally be argued to evidence representations’ utility. Kuhn makes a similar point about the frustrations of searching for explicit and well-defined “rules” for a paradigm:

Recognizing that frustration, however, makes it possible to diagnose its source. Scientists can agree that a Newton, Lavoisier, Maxwell, or Einstein has produced an apparently permanent solution to a group of outstanding problems and still disagree, sometimes without being aware of it, about the particular abstract characteristics that make those solutions permanent. They can, that is, agree in their identification of a paradigm without agreeing on, or even attempting to produce, a full interpretation or rationalization of it. Lack of a standard interpretation or of an agreed reduction to rules will not prevent a paradigm from guiding research. (Kuhn, 1962/2012, p. 33)

Moreover, despite the tendency to frame the conflict between the enactive and cognitivist camps in terms of paradigms, enactivists also tend to be forthright about what they are up against in this regard: “[cognitivism] is a successful formula for the practice of research, a powerful recipe for generating empirical hypotheses and testing them, accumulating results, and assembling theoretical frameworks. It is the way things are currently done in the sciences of the mind, the way they have been done for over six decades” (Di Paolo et al., 2017, p. 2).

Given the absence of a looming crisis within cognitivism itself,^2^ an extra step has, it seems, had to be added to the enactive battle plan. First, enactivists have tried to offer a compelling case to cognitivists that there *already exists* an intractable problem within the cognitivist paradigm that will eventually lead to its downfall, a ticking time bomb waiting to go off inside cognitivism. That is, enactivists have the additional challenge of stoking awareness of perceived deficiencies, those that will at some point turn into intractable problems within the cognitivist paradigm itself, and which enactivism is better equipped to handle. This is what we call the *hard sell* of enactivism.

The most prevalent way enactivists have sought to convince cognitive science of this impending breakdown is by proposing an oversight in the cognitivist framework, the existence of some sort of unbridgeable *explanatory gap*. For example, Thompson (2007) points to an explanatory gap between cognitive processes and consciousness in cognitivism, popularized as “the hard problem of consciousness” in Chalmers (1996), proposing that “the enactive approach offers important resources for making progress on [this] explanatory gap” (p. 14). However, this turns out to be a bit of a false start: he also concedes that some cognitivists (Pylyshyn, 1984) have dismissed this problem as not “within the province of cognitive science” (Thompson, 2007, p. 6). In order for this to be a problem that poses the sort of threat to cognitivism that would lead to a paradigm shift, one first has to accept that this largely philosophical problem translates to a tractable empirical problem that cognitive science wants to solve, that is, to place the gap in cognitivism as seen by cognitivists themselves. While certain cognitivists may be keen to investigate consciousness and even consider it a crucial target for explanation, not doing so—or outright denying the relevance of consciousness to cognitive science—does not bring a cognitivist to ruin within their own paradigm.

Similarly, we find an explanatory gap alluded to in Di Paolo et al. (2018), but here the target is functionalism as a cognitivist explanatory methodology. They argue that while cognitivists have embraced embodiment as an explanatorily relevant factor in studying cognition, this embrace has simply amounted to “a larger causal structure for building functionalist explanations” (p. 19). That is, cognitivist assimilations of embodiment still leave out the interactive nature of the agent-environment relationship. Di Paolo et al. argue that “enactive ideas also permit theorizing about concrete bodies, bodies that live and labor, suffering bodies, subject to disease, pains, and joys, and that must attend to needs for food, shelter, sex, relating to others, and so on” (Di Paolo et al., 2018). However, functional explanations provided in cognitivist theories of niche construction, cognitive offloading, and social cognition can be considered interactive, though perhaps transactional rather than recursive, as enactivists tend to conceive of these processes. A functionalist already has to find this characterization lacking in order to need an alternative. It is also not clear in what way functional explanations fail to permit theories of our basic bodily needs—this is precisely the methodology of basal cognition research, for instance (Lyon et al., 2021). And again, one must be convinced not only that cognitivism is incapable of providing theories for these issues, but also that these issues are within the purview of cognitive science to begin with.

It does not seem as though any major explanatory gaps are threatening cognitivism. From within cognitivism, enactivism poses no major threat by adding explananda in need of explanantia that simply do not exist within the cognitivist framework. This is a misguided criticism, akin to claiming a car does not work properly because it does not fly—such an observation does not reveal cognitivism to be a lemon. To demonstrate that both cognitivist and enactivist theories are capable of providing explanations for phenomena that meet the criteria as defined from *within* their own research programs, we will look at the well-known example of social cognition.

### Social cognition: Having it both ways

Both enactivism and traditional cognitive science have a deep interest in how it is that people are able to understand and predict the behavior of others. Here we take a typical example of how cognitivist and enactive explanations are pitted against one another using competing explanations of social cognition. The mainstream cognitivist account—sometimes called the “mindreading” account—holds that social cognition consists in the ability of people to infer and attribute “beliefs, desires, emotions, and intentions” (Spaulding, 2018, p. 1) to others in order to make sense of and predict their behavior. The specific means by which people are able to make these attributions is generally thought to involve either theorizing about or simulating the contents of another agent’s mind, and thereby inferring their reasons for acting. These two proposals are known, respectively, as theory theory (TT) and simulation theory (ST). While they differ on the details of how mindreading gets done, both assume that mental states and the content they bear are central to the task of understanding others’ beliefs, intentions and so on. Since we are never in direct contact with the mental states of others, we must use these sophisticated capacities to mindread at a distance, either by inferring the inner mental state of another person through folk psychological rules and norms, or by taking on and simulating their mental states so as to understand their behaviors (Spaulding, 2018).

One enactive account, interactionist theory (IT), rejects these core assumptions and assumes instead that most social cognition involves the direct perception of the minds of others—no mindreading required. According to IT, social cognition does not fundamentally involve inference to others’ hidden internal mental states and beliefs (Gallagher, 2008). Rather, we employ strategies of inference infrequently, in more complicated situations; this is the exception, not the rule. Social cognition is constituted in interaction with other agents, where mental states and intentions are directly perceived as embodied processes. This kind of interactive sociality can be broken down into two kinds: primary intersubjectivity, which involves a very basic and direct perceptual understanding of another person (happy, restless, about to get up), and secondary intersubjectivity, wherein we understand the intentionality of the other in relation to a shared context (looking at, gesturing towards, feeling about; Trevarthen and Hubley, 1978).

One way of demonstrating differences in commitments, empirical design and evaluation of the two accounts is by comparing their interpretations of the false-belief test, a staple of social cognition research that aims to demonstrate the development of theory of mind capacities in young children. A standard version of the task involves dolls acting out a story. The doll in the story puts their toy down in a location and then leaves the room. While the first doll is out of the room, a second doll moves the toy to another location. The first doll returns, and the child watching the story is asked where they think that first doll will look for their toy (Onishi and Baillargeon, 2005). The results show that children under 4 years old typically respond by saying the character should look for their toy in the new location it was moved to, suggesting that they are unable to understand that the character could not possibly know the toy had moved. At age four and up, children correctly indicate that the character will hold a false belief about the location of the toy, thinking it is still where they left it. The younger children, it is held, are unable to distinguish their own beliefs from the beliefs other agents would possess. The ability of more developed 4 year olds to successfully predict that the character will hold a false-belief is taken to demonstrate a capacity to attribute beliefs to other agents, especially beliefs that differ from their own.

Mindreading theorists account for this result with the explanation that younger children are missing a discrete mindreading capacity. Around age 4, children gain (through some combination of sociocultural and biologically driven development, the exact mix depending on whether you ask TT or ST theorists, and which theorists in particular) the capacity to infer the contents of another’s mind. Hence social cognition is explained as an inferential and interpretive capacity, an interpretation that has been well-supported by numerous empirical studies (Spaulding, 2018). The empirical findings that are taken to support mindreading accounts of false-belief tests have been shown by meta-analysis to be highly reliable and consistent with one another (Wellman et al., 2001). Further, in the case of ST, research into neural mechanisms of simulation (such as mirror neurons) has been argued to bolster the physical plausibility of the account, and integrate the psychology-level phenomenon with the neuroscientific facts (Goldman, 2006).

However, if we assume, as IT does, that primary and secondary intersubjectivity establish the core of what happens in social cognition, then it is not surprising that children fail at the more advanced kind of social cognition required by the test. The capacity to express to another person the emotions and intentions that are directly perceived involves the development of sociocultural skills of expression. Young children perform poorly on the false-belief test compared to older children and adults not because they lack social cognitive abilities, but because the experimental design is not actually eliciting a demonstration of ordinary social cognition (Gallagher, 2008). The older children succeed at the task because they have had more time to be properly encultured with the social and linguistic norms of giving reasons for the actions of others, not because they possess a mindreading ability that they did not before (Gallagher and Hutto, 2008).

### Comparing the explanations

So, how has this interactionist alternative been received over in the mindreading camp? If the enactive account is explanatorily superior, we might expect that advocates of TT and ST would question their adherence to the cognitivist paradigm. On the contrary, prominent defenders like Spaulding and Carruthers are unimpressed with IT, which in their view has very little meat on its bones when it comes to properly explaining social cognition. Spaulding (2015, 2018) argues that using primary and secondary intersubjectivity to investigate social cognition relies too heavily on phenomenological evidence. By her lights this is insufficient since under cognitivism, social cognition can be performed sub-personally and so be inaccessible to the agent on a conscious level.

Carruthers (2016) also dismisses the interactionist story. In the face of alternative interactionist explanations, Carruthers simply says: “…these approaches have not proven empirically fruitful: no new discoveries have resulted. And in every case where a determinate proposal of this sort has been tested and controlled for, it has been refuted” (Carruthers, 2016, p. 142). No new empirical data of relevance has been gleaned from interactionism, and so, for Carruthers, “no new discoveries have resulted” (Carruthers, 2016). Cognition is something that goes on in the head, and behavior is the consequence of that head-based activity. Carruthers holds to this view in spite of the existence of research investigating the embodied aspects of social cognitive phenomena such as autism (Torres et al., 2013; Hobson, 2014) and gesture in communication (Goldin-Meadow, 1999; Cole et al., 2002). A description of social cognition that does not build towards an explanation of how social cognition is done in the head is, for Carruthers, not addressing the appropriate explanantia, and so in his view the enactive story does not just present a bad explanation, *it does not provide any explanation of social cognition at all*.

So, as Di Paolo et al. (2018) observe regarding the mindreading mainstream: “the epistemic frame remains untouched by experimental observations that could otherwise be used to examine it. The question ‘If participants do not make inferences, what else could be going on?’ is not given a proper shot.” (p. 16) But is Carruthers wrong to operate from within such an exclusive framework? We do not see any immediate reason to think so. Both cognitivists and enactivists have their own distinct ideas about what social cognition is and consequently what features of the world should be the target of an explanation thereof. Clearly those ideas are quite different. Based on the discussion so far, there would be little reason to adjudicate that one side deserves, just on the strength of its interpretation, to sweep the field and knock over the opposition.

However, Hutto and Myin (2013) have proposed a criterion for demonstrating the superiority of one idea over the other: the best explanation is one that offers empirical adequacy, which we take to imply that the explanation offers more than, and in doing so demonstrates the *in*adequacy of, the competing theory. In trying to conceive such a means of explanation-comparison, a challenge arises that is simple to understand but extremely difficult to solve. The two sides here have basically outlined the problem already: what counts as the best explanation fundamentally depends on the assumptions you already have about what cognition is. To stick with our example, enactivists believe that basic social cognition is non-representational, while mindreading accounts are committed to representationalism. For the latter camp, cognition is a mostly brain-bound, internal event, not something constituted in tandem with behavior. Meanwhile, enactivists consider the environment and entire organism (not just the brain) to be fundamentally constitutive of cognition, not merely causal inputs and outputs for the brain. Empirical adequacy, and hence the better explanation, is very much in the eye of the beholder. There is no room for arbitration here: Carruthers thinks minds are representation-manipulating devices which make inferences at a distance about other such devices and so these items must appear in an explanation, non-negotiably, because those are the required explanantia according to the cognitivist framework.

Hutto and Myin likewise argue that their assumptions are the correct ones. So whose assumptions are correct? An immediate hurdle here concerns the nature of background assumptions in science: the raw data of cognitive science systematically underdetermines which hypothesis we should accept about cognition, and what fills this gap between data and theory are the assumptions research communities are committed to. This has been discussed at length in the philosophy of science literature:

“…how one determines evidential relevance, why one takes some state of affairs as evidence for one hypothesis rather than for another, depends on one's other beliefs, which we can call background beliefs or assumptions. Thus, a given state of affairs can be taken as evidence for the same hypothesis in light of differing background beliefs, and it can be taken as evidence for quite different and even conflicting hypotheses given appropriately conflicting background beliefs. Similarly, different aspects of one state of affairs can be taken as evidence for the same hypothesis in light of differing background beliefs, and they can serve as evidence for different and even conflicting hypotheses given appropriately conflicting background beliefs.” (Longino, 1990, p. 43)

Hence the good sense in Gallagher’s (2017) claim that we should not expect “that there could be one single critical experiment that might decide the issue between the representationalist and the enactivist” (p. 21). Carruthers and Spaulding are in the right in saying that enactivist assumptions do not force a shift in a cognitivist’s opinion on the best explanation for cognition. Researchers both scientific and philosophical typically view the targets of their field through a set of interconnected background beliefs established during their training into a particular research paradigm, through the lens of which they judge data, hypotheses, and theories. For instance, when outlining Gallagher’s (2004) account of primary intersubjectivity, Spaulding attempts to clarify Gallagher’s claim that mental states are directly perceived through bodily activity by stating that “[p]rimary intersubjectivity is more like *bodyreading* than mindreading” (Spaulding, 2010, p. 122). But this is precisely what Gallagher is trying *not* to say here; Gallagher is making the point that the mind is present in bodily activity, so describing this as bodyreading misses the point. Almost reflexively it seems, Spaulding forces primary intersubjectivity into the inferential mold.^3^

Similarly enactivists will never concede to Carruthers’ explanation, because they do not think representations are involved in basic social cognition, and so reject inferences by mental mechanisms as the appropriate explanation for social cognition. The two opponents simply have no way to neutrally evaluate their respective explanations from some perspective outside of these commitments. And, neither side has a way to defeat the background beliefs of the other. Both theories are empirically adequate for those who hold the assumptions of their paradigm.

For cognitivists, cognition can be fully explained in the established fashion. They have set out their idea of what cognition is, where it is, and what an explanation of it should look like. There is coherence between these ideas; based on what cognitivism proposes cognition is, their explanatory strategy and goals make sense. Put another way, the explanatory gap is not a problem for cognitivism, since in the main cognitivists do not see subjectivity as explanatorily relevant to how cognition works. It simply is not a problem that must be solved in their view. And, similarly, the cognitivist must concede that if cognition is indeed a process spanning brain, body and environment that is deeply intertwined with questions of agency and subjectivity, then the enactive explanatory strategy is coherent with that position. A full explanation on this enactive view could neither begin nor end with physical and mental states happening in the brain.

In these sections we have addressed the hard sell of enactivism: the claim that enactivism unveils an irresolvable explanatory gap within cognitivism, and as such generates a crisis in cognitivism that will lead to a paradigm shift. We showed that this is not the case either in theory or in practice. First, there is no reason to think there is a gap within cognitivism that will bring about a paradigm shift, nor that enactivism is the harbinger of such a collapse. Second, the alternative view that enactivism provides better explanations than cognitivism, and therefore ought to replace it, does not bear out either. The social cognition literature provides a perfect example of how explanations are taken to be better based on previously existing commitments, as well as the resilience and adaptability of the cognitivist paradigm to integrate insights from enactivism (though in their own terms). Enactive interpretations of a given phenomenon aren’t exactly up to the task if the goal is to displace or defeat the cognitivist interpretation, and vice versa. With the hard sell unlikely to shift any paradigms, this leaves us with the *soft sell*.

## The soft sell

The soft sell of enactivism promotes the attractiveness of an enactive framework in contrast with mainstream cognitivism. This contrasts with the hard sell: the emphasis of the soft sell is on the strong desirability of enactivism, rather than asserting its explanatory superiority according to some set of criteria. Faced with an entrenched cognitivist paradigm that permeates from cognitive science right down to popular folk understanding of the mind, the soft sell instead presents the allure of enactivism to potentially interested philosophers and scientists. In this section we elaborate on this soft sell of the enactive paradigm: that if researchers would just entertain some enactivist assumptions, they will then see the value in enactive explanations. Given the background assumptions that go into theory choice, we detail some of these commitments that might lead one to find enactivism more enticing than cognitivism.

One such appeal suggests that enactive explanations should be more attractive than their cognitivist alternatives because they are more *conceptually elegant* (Hutto and Myin, 2017). Conceptual elegance here appears to mean an account free from the unnecessary conceptual bloat that talk of internal representational and symbol-manipulating reasoning processes introduces into explanations of cognition. Cognitive phenomena are more elegantly explained when an account can be given without assuming the need for these kinds of entities, and enactivism provides the resources for doing precisely this. For example, Hutto (2019) demonstrates his commitment to conceptual elegance in describing his strategy for removing unneeded entities from an account of mathematical reasoning in order to build an enactive alternative: “[s]ubtract any residual commitment to mental representation, information-processing stories, and neuro-fetishism…Subtract any residual constructivism, anti-realism, and idealistic elements from the account. Finally, subtract any lingering psychologism about mathematics and its content” (Hutto, 2019, p. 835).

Taking issue with various-isms is here a running theme: what Hutto calls *neuro-fetishism*, *residual constructivism*, *psychologism*. While we can trace Hutto’s problems with these ideas back to the common denominator of the hard sell—they are all representationalist accounts that are meant to be explanatorily worse than the enactive alternative—the soft sell seems to go beyond that concern. Describing the outward signs of representationalist commitments as a kind of *fetishism* is certainly evocative and seems to voice a certain distaste; likewise *psychologism* stirs up ideas of a dogmatic scientism. Similarly, the accusation of *intellectualism* (Hutto and Myin, 2013) is presented as a distinct criticism involving a concern over an unattractive mode of thinking that languishes in early modern and Platonic notions of humans as chiefly *thinkers*, rather than *doers*. While this could all be read as merely a colorful extension of the hard sell, we see a distinct strategy here: Hutto’s characterization of the unnecessary and problematic clutter of cognitivism appeals to the more parsimonious or ontologically minimalist qualities of enactivism, rather than appealing to its offering a necessarily (or perhaps simply measurably) superior explanation.

Taken by itself, though, this does not provide a particularly forceful case for the paradigm-shifting nature of enactivism. That enactivist explanations are not cluttered with mental entities such as states and representations may be alluring to those with a preference for desert landscapes, but preferences alone cannot upset a dominant paradigm. Tastes may factor into the choice of best alternative theory *during* a Kuhnian crisis, but paradigms do not rise and fall due to arbitration over who is the greater aesthete.

Another soft sell strategy we see employed by enactivists is to marshal their efforts toward the *prima facie*, immediately observable features of nature. In their view, these provide powerful evidence that cognition is constituted by the organism and environment through their conjoined dynamical interactions, and not through internal computations. It is only the inattention, deliberate or innocent, of the cognitivist mainstream to these features of the world that hides the facts which corroborate with the enactive picture. When properly reflected upon, computational explanations “to many people, do not match well the situated and richly context-dependent experiences and activities they enact every day” (Di Paolo et al., 2017, p. 12).

Hutto and Myin propose that part of remedying this inattention is to take a clear-eyed look at cognition, unencumbered by assumptions about the kinds of representational and computational activity that cognition is typically thought to require, in order to reveal the relationality that we experience first-hand out in the world: “In rejecting [cognitivism] in this domain, [enactivism] takes *at face value* what attending to the architectonic details of how these agents work suggests: that the specified bodily and environmental factors are equal partners in constituting the embodied, enactive intelligence and cognition of these artificial and natural agents” (Hutto and Myin, 2013, p. 44). A clear-eyed look at cognition, taking the world “at face value” as they put it, will reveal that these details of the organism-environment coupling are vital parts of the explanatory story for cognition. Neglecting them means neglecting the very foundations of cognition.

A similar line is taken by Di Paolo et al. (2017): “The assumptions that validate specific lines of investigation in this mode are not universal though they are treated as if they were, particularly the assumption that the mind works like a special kind of computer. *A careful look* at what we know about our bodies, about their biology, and the way they organize themselves into powers and sensitivities, a look at the way we experience the world as situated creatures in complex relations to other creatures tells a rather different story” (Di Paolo et al., 2017, p. 2; emphasis added). This lack of neutral observation has “biased cognitive explanations toward the disembodied and intellectualist end of the spectrum, the kind of explanations that, to many people, do not match well the situated and richly context-dependent experiences and activities they enact every day” (Di Paolo et al., 2017, p. 12). On this view, if we take a more careful look at living cognitive organisms we will see that they give us no obvious signs of operating, at least at the level of basic cognition, as representation-hungry symbol-manipulators.

We do not here take issue with the enactive interpretation of the kinds of “face value” facts about cognition that enactivism tries to capture. After all, any novel scientific theorizing is going to have to originate with people seeing something new in observations of nature. But is it actually obvious to a clear-eyed observer that computationalism misses features of life and mind that are plain to see for the unencumbered? We take it as fairly given in contemporary philosophy of science and epistemology that human beings are always embedded in some context that flavors their interpretation of events, scientists included. Scientists are embedded in historically situated communities that offer and constrain possible interpretations of phenomena (Kuhn, 1962/2012). Our appraisals of incoming observations of nature are necessarily made in the context of a web of belief, and in this holistic way our background beliefs and assumptions both allow us to make sense of events and accommodate them into our existing understanding of the world (Quine, 1951).

With this in mind, it is hard to see how a cognitivist appraisal of some cognitive phenomenon is less clear-eyed or less neutral than the appraisal made by an enactivist. It also seems hard to argue that an enactivist is uncommitted or pure of mind. Enactivism is, if anything, a novel *lens* through which to interpret the phenomena of cognition, not the absence of a lens.

In this section we have argued that the soft sell of enactivism, like the hard sell, does not herald a coming paradigm shift. Arguments that enactivism is simply evident to a careful, neutral, or unencumbered observer do not quite hit the mark, and appeals to esthetic or other pre-existing partialities do not offer a rigorous way of demonstrating that the enactive account of cognition is explanatorily superior to the cognitivist account. Cognitivism is only deficient if one abandons the background assumptions that motivate cognitivism, and one is not given a good reason to abandon those assumptions by the successes of enactivism. This may be a bitter pill to swallow for some, but the alternatives are either to embark on a quest to solve the problem of underdetermination, or show that there is an alternative way to argue that enactivism is preferable to cognitivism. To those who choose the former, we wish them well; as for the latter, this will be our focus in the remainder of the paper.

## From paradigm to philosophy of nature

A paradigm shift is off the table. No internal strife is collapsing the cognitivist consensus, and the adequacy of enactivism is relative to the differing background assumptions that motivate it, which do not bode anything for cognitivism. But a shift in paradigms need not be the endgame for enactivism anyway. Championing a paradigm shift sells short the enactive project by limiting its scope to a single research program. Instead, what enactivism offers is something more integrative, more broad, and more fundamental: a *philosophy of nature*.

The notion of a philosophy of nature used here originates with Godfrey-Smith’s (2001) discussion of developmental systems theory (DST). DST, for context, is a “general theoretical perspective” on many of the big issues in biology, such as “development, heredity and evolution” (Oyama et al., 2001, p. 2). It places a strong emphasis on holism in explaining and understanding biological phenomena, and sets itself up in opposition to the more genetically-deterministic mainstream within biology. While DST has had a strong appeal for many theorists, its exact nature, and its exact aims, remained unclear to non-enthusiasts, as expressed by Godfrey-Smith: “What kind of theory is DST? Is it a scientific theory or a philosophical theory? Is it an empirical hypothesis, a suggested program of research, a philosophical gloss on our existing knowledge, or what? What difference does it make whether or not the central ideas associated with DST are true?” (Godfrey-Smith, 2001, p. 283).

In light of this confusion about what DST provides, Godfrey-Smith proposes a distinction between a *scientific research program* and a *philosophy of nature*, saying that what DST offers is a mixture of the two. A research program must offer guidance for empirical work, though this can vary in specifics between programs. He points out that DST includes a set of foundational empirical claims, foundational conceptual language, and explanatory standards (Godfrey-Smith, 2001). These are consistent with what we would find in a research paradigm, as described above and by Godfrey-Smith elsewhere: “… a paradigm, in Kuhn’s theory, is a whole way of doing science, in some particular field. It is a package of claims about the world, methods for gathering and analyzing data, and habits of scientific thought and action” (2003, p. 77). A philosophy of nature, on the other hand, “can use its own categories and concepts, concepts developed for the task of describing the world as accurately as possible when a range of scientific descriptions are to be taken into account, and when a philosophical concern with the underlying structure of theories is appropriate” (Godfrey-Smith, 2001, p. 284). A philosophy of nature is said to come “after” science in the sense that it redescribes the work of one or more research programs in an effort to integrate their findings into the most accurate picture of the world.

Godfrey-Smith explains the relationship between a philosophy of nature and a particular science as follows: “When doing philosophy of nature, we are trying to understand the universe and our place in it. The science of biology becomes an instrument—a lens—through which we look at the natural world. Science is then a resource for philosophy rather than a subject matter” (Godfrey-Smith, 2014, p. 4). Where the paradigm is intended to guide empirical research within a specific field, a philosopher engaging with a philosophy of nature “will [instead] look at how the message of one part of science relates to that of another, and how the scientific view of nature relates to ideas we get from other sources” (Godfrey-Smith, 2014, p. 4). The philosophy of nature generates questions about what the paradigm takes for granted, and examines problems in describing the world that appear as inconsistencies in disparate paradigms, attempting to put together “an overall picture of the world” (Godfrey-Smith, 2014).

The idea that enactivism might likewise be thought of as a philosophy of nature was first proposed in print by Gallagher (2017).^4^ Gallagher presents the option of treating enactivism as a philosophy of nature as a solution to the problem of holism, to the effect that the more holistic the perspective on cognition, the more appropriate it is to treat that approach like a philosophy of nature. This is meant to be the case because “it is difficult to operationalize holism. Neither experimental control nor the division of labor in science allows for all factors to be taken into consideration at once” (Gallagher, 2017, p. 21). However, this does not mean that enactivist concerns are merely “explanatorily idle debates” (Miłkowski et al., 2018, p. 11). The stakes of a philosophy of nature are perhaps higher than those of any specific research program, a point made salient through Gallagher’s “clunky robot” problem:

“[J]ust as one can design a robot by assigning teams to construct different modules, which turn out to work well as individual modules, it may happen that when the modules are brought together, they don’t play well together. No one has considered the relational aspects of how one module will dynamically connect with another in a complex system, and the result is a clunky machine-like behavior. The same problem can be found in theory construction. Scientific experiments, designed within the framework of their own particular paradigm, often study the pieces of a system but don’t always consider how the dynamical relations among those pieces work, and don’t always have the vocabulary to address those relations.” (Gallagher, 2017, p. 22)

Gallagher’s analogy makes clear that the stakes of theory building do not merely concern the ability of a research program to form and solve problems and generate data. Integration between research programs, especially in an interdisciplinary research area such as cognitive science, is a necessary consideration for making progress.

In this way, a philosophy of nature ought not to be thought of as only coming after science, but also as theoretically underpinning scientific progress. Where a paradigm is a focused scientific research programme in which a narrow set of shared beliefs are a boon for facilitating phenomenon-specific methodologies, tools, and language for data interpretation and synthesis, a philosophy of nature consists of describing (or re-describing) these in terms of overarching beliefs that line up with a specific ontological picture of the world. In this way, a philosophy of nature might be thought of more as a worldview. That is, its commitments are not paradigm-specific, but structural: they provide the basis on which we can build the narrow sets of shared beliefs that support a research program, and further, they underpin and support the integration of research programs.^5^

This supports the kinds of possible outcomes that Godfrey-Smith has proposed: “This philosophical work might well come to have an effect on the science itself; it might change the hidden or overt philosophical commitments of the scientists. But the absence of such an effect on science does not rob the philosophical work of its value” (Godfrey-Smith, 2001, p. 285). A philosophy of nature does not necessarily need to influence science, and certainly there are plenty of philosophies of nature that make no effort to do so. Importantly, this sets an enactive philosophy of nature apart from both the hard and soft sells. Building a philosophy of nature involves developing an account of life and mind that is philosophically defensible and sensitive to what the relevant sciences are up to, but it does not require or entail selling enactivism as a paradigmatic rival to cognitivism.

However, a philosophy of nature is oftentimes an impetus for scientific advancement, as it provides the theoretical grounding on which a research paradigm can be built. We have discussed in the previous section the need for taking background beliefs into account in thinking about how it is that there can be alternative paradigms which are both capable of producing problems and solutions. Philosophies of nature form the grounding for the alternative theories proposed during a scientific revolution, when a dominant paradigm has a crisis due to internal flaws and inconsistencies. Were there no philosophies of nature, there would be nothing on which to develop these new paradigms. Thus, we agree with Gallagher that “even if enactivism were to be considered a philosophy of nature, it would not be right to conclude that it cannot offer concrete hypotheses or raise novel scientific questions” (Gallagher, 2017, p. 24). The issue is not whether enactivism might be capable of doing so, but the difficulties therein.

Of course, enactivism is not a monolithic or unified philosophy of nature. There are varying and even hybrid versions of enactivism (Ward et al., 2017). However, all varieties reject the view that cognition is computational, as well as the focus on the brain and central nervous systems as the locus of cognition: “[i]nstead of understanding cognition as a *computer-like* process, enactivism starts by considering it as a *lifelike* process anchored in the living body” (Di Paolo et al., 2017, p. 20, emphases original). However, not all cognitive scientists are committed to a computational ontology of cognition. Computational terminology retains its practical and heuristic value so long as it is explanatorily useful within its research program. What Gallagher points out is that as a philosophy of nature, enactivism is after much more than a replacement of terminology in cognitive science; enactivism’s ontological commitments demand an understanding of the explanatorily relevant phenomena of cognition—being irreducible, processual, embodied, and relational—that is incompatible with the cognitivist research program. On his view, progress in cognitive science is being held up by its ongoing commitments to the computational metaphor:

“Being a pragmatist about the vocabulary of representation…or about the vocabulary of inference, is at best only a temporary stance toward a set of placeholders that need ultimately to be cashed out not just in a different conception of brain function, but in a different philosophy of nature. An alternative way of thinking about nature should push hard on cognitive scientific practice in a way that makes doing science more difficult, but also more productive.” (Gallagher, 2017, p. 126)

What enactivism *does* do is present a set of background beliefs that make traditional operationalizations of cognition problematic. There is nothing to suggest enactivist operationalisations are inherently trickier to execute. They simply fly in the face of conventional dogma about what minds are, where they begin and end, how they can be broken down into examinable components, and the like. But this is only one aspect, or consequence, or having a different worldview. We have discussed the nature of background beliefs and assumptions, and how these weave an interconnected web of ideas that encourage and constrain our hypothesizing. They allow us to bundle together data into intelligible hypotheses, to determine our epistemic standards, and speak as a research community about the kinds of accounts of phenomena we find satisfactory or lacking. In a discipline that requires a great deal of conceptual work, the guiding conceptual compass for research is the web of beliefs and assumptions that make up a philosophy of nature—and a compass is used to tell us where we ought to be heading, not where we are.

Other enactivists appear to concur with this assessment. Representations are “an awkward place-holder for an explanation that still needs to be given in dynamical terms of an embodied, environmentally embedded, and enactive model” (Gallagher, 2017, p. 106), and cognitivist attempts to explain seamless, pragmatic bodily interactions with the world in representational terms “always miss something that cannot be expressed as the summation of several rules, objective standards and precise norms” (Di Paolo et al., 2017, p. 12). Hutto and Myin (2013) take to task even those enactivists who they see as “slipping into unguarded talk of perceivers’ (or their brains’) making assumptions, predictions, and judgements in ways that look decidedly as if the view is committed to the existence of propositional rather than essentially practical knowledge” (p. 26). The stakes are not limited to the best way to generate data for understanding specific problems about cognition. Enactivist concerns range far beyond the boundaries of a paradigm.

## Conclusion

We have identified that enactivism contains two very distinct projects: a critique of cognitivism, and the building of its own alternative. Though enactive work so far has (as we have shown in this paper) assumed their mutual entanglement, in fact the latter is not substantially assisted by the former, and in any case the task of dismantling cognitivism has for all practical purposes been unsuccessful. Whether or not cognitivism will eventually be replaced remains to be seen, but if that time comes, enactivists need to have done more than act as harbingers of the revolution: actually existing enactivism has to be a viable alternative. If enactivism is as Wheeler (2017) says akin to a revolutionary manifesto, then let us stick with the metaphor for a moment and consider that the step between manifesto and successful revolution is praxis.

Our strategy in this paper has been to clear out the paradigm talk that has cluttered the eyeline of enactivist work, and setting aside the drive to defeat cognitivism that has obscured the real horizons of the enactive project. Neither the hard nor soft sells of enactivism have been a success. It would seem that enactive philosophy is not steering mainstream cognitive science in a new direction. Science will continue its work so long as the dominant paradigm keeps enabling research. Enactivist work skewed towards rooting out cognitivist corruption, and contrasting the philosophical virtues of their own framework with the flaws of cognitivism, threatens cognitivism with no more than a heuristic reminder to pay attention to the interactive aspects of cognition (Miłkowski et al., 2018).

This raises a question: what if, as enactivists argue, cognitivism eventually stalls as a paradigm, what has enactivism offered in terms of grounding for an alternative? Keijzer (2001) has warned that “[w]ithout a good replacement, even a feeble story will remain the best explanation available (Stich, 1991). Without a ‘Copernican alternative’ we will not have a ‘Copernican turn’” (pp. 44–45). Thus far, enactivism does not have a comprehensive story for how the various capacities of the mind can be explained, and how these can be examined empirically. Part of the problem here is undoubtedly due to the shaky conceptual ground of enactivism: there still is no overarching framework, nor do we see the kind of scientific “divide and conquer” schemes for operationalisation taken up in cognitivist cognitive science (we think here of Marr’s (1982) framework for perception research, Gibson’s (1979) ecological approach, and Craver’s (2007) mechanism as this kind of work). These are frameworks that allow for the operationalisation of theory, and with the explicit goal of establishing explanatory goals and norms for a field.

Nevertheless, there are enactivists working to expand the capacities of enactivism. In some corners of science and philosophy, the fields are being tilled and the careful work of building an enactive account is being carried out. For philosophers, the steady grassroots of this work consists in building the most accurate picture of nature that enactivism can provide, furnishing the concepts that will allow us to understand this new image, and which ultimately will work to integrate the questions and findings of diverse fields of scientific study that are the subject matter of enactivism.

## Author contributions

RM and NB jointly conceived and wrote the paper. All authors contributed to the article and approved the submitted version.

## Funding

NB received support through the Templeton World Charity Foundation Grant: “Intelligent Agency on Multiple Scales” (TWCF0463) and a PERL grant (Prioritising Emerging Research Leaders) at the University of Wollongong.

## Conflict of interest

The authors declare that the research was conducted in the absence of any commercial or financial relationships that could be construed as a potential conflict of interest.

## Publisher’s note

All claims expressed in this article are solely those of the authors and do not necessarily represent those of their affiliated organizations, or those of the publisher, the editors and the reviewers. Any product that may be evaluated in this article, or claim that may be made by its manufacturer, is not guaranteed or endorsed by the publisher.

## Footnotes

^1^Not all enactivists are strictly anti-representational. Hutto and Myin (2017) (see also Myin and van den Herik (2020)) have recently argued that certain linguistic capacities involve "representations"—though in both accounts it is unclear how the act of representing through language implies the necessity of representational cognitive processes. Such accounts are overly promiscuous in what counts as a cognitive process—we find this to be an unnecessary concession in conflict with core enactivist commitments.

^2^This is not to say that such a crisis is impossible, and nor will we necessarily have ample advanced warning of it. Nevertheless, we have seen that even in the wake of the "replication crisis" which hit psychology in recent years that researchers identify methodological failings resulting from sociological factors as causes of this crisis, not conceptual flaws in cognitivism (i.e., Earp and Trafimow, 2015). This inclines us to think that cognitivism remains fairly stable for the time being.

^3^Demonstrating a similar process, Schönherr and Westra (2019) show in detail how an interactionist story—which elevates the interaction itself to an ontologically and epistemically distinct feature of cognition—can be interpreted into a coherent cognitivist story.

^4^Gallagher credits this insight to Cecilia Heyes.

^5^Heras-Escribano (2021) has argued that ecological psychology offers both a philosophy of nature and a well-developed research program, and thus is at an advantage to enactivism. We do not here offer a comparison of enactivism and ecological psychology, and we do not necessarily disagree. However, we note that Heras Escribano’s contention is with the idea that a philosophy of nature redescribes data produced by other research programs (Godfrey-Smith, 2001; Gallagher, 2017). We argue here that a philosophy of nature does more than that.
